# Genome-wide Profiling of RNA splicing in prostate tumor from RNA-seq data using virtual microarrays

**DOI:** 10.1186/2043-9113-2-21

**Published:** 2012-11-26

**Authors:** Subhashini Srinivasan, Arun H Patil, Mohit Verma, Jonathan L Bingham, Raghunathan Srivatsan

**Affiliations:** 1 Institute of Bioinformatics and Applied Biotechnology, Biotech Park, Electronic City Phase I, Bangalore, 560100, India; 2 Present address: Pacific Biosciences, 1380 Willow Rd., Menlo Park, CA, 94025, USA

## Abstract

**Background:**

Second generation RNA sequencing technology (RNA-seq) offers the potential to interrogate genome-wide differential RNA splicing in cancer. However, since short RNA reads spanning spliced junctions cannot be mapped contiguously onto to the chromosomes, there is a need for methods to profile splicing from RNA-seq data. Before the invent of RNA-seq technologies, microarrays containing probe sequences representing exon-exon junctions of known genes have been used to hybridize cellular RNAs for measuring context-specific differential splicing. Here, we extend this approach to detect tumor-specific splicing in prostate cancer from a RNA-seq dataset.

**Method:**

A database, SPEventH, representing probe sequences of under a million non-redundant splice events in human is created with exon-exon junctions of optimized length for use as virtual microarray. SPEventH is used to map tens of millions of reads from matched tumor-normal samples from ten individuals with prostate cancer. Differential counts of reads mapped to each event from tumor and matched normal is used to identify statistically significant tumor-specific splice events in prostate.

**Results:**

We find sixty-one (61) splice events that are differentially expressed with a p-value of less than 0.0001 and a fold change of greater than 1.5 in prostate tumor compared to the respective matched normal samples. Interestingly, the only evidence, EST (BF372485), in the public database for one of the tumor-specific splice event joining one of the intron in KLK3 gene to an intron in KLK2, is also derived from prostate tumor-tissue. Also, the 765 events with a p-value of less than 0.001 is shown to cluster all twenty samples in a context-specific fashion with few exceptions stemming from low coverage of samples.

**Conclusions:**

We demonstrate that virtual microarray experiments using a non-redundant database of splice events in human is both efficient and sensitive way to profile genome-wide splicing in biological samples and to detect tumor-specific splicing signatures in datasets from RNA-seq technologies. The signature from the large number of splice events that could cluster tumor and matched-normal samples into two tight separate clusters, suggests that differential splicing is yet another RNA phenotype, alongside gene expression and SNPs, that can be exploited for tumor stratification.

## Background

Until recently, the extent of RNA diversity resulting from alternative splicing had been consistently underestimated. In the early 90s, researchers projected that only 5% of the human genes were alternatively spliced based on PCR methods, which was revised upward to 35% by the end of the decade using mining EST database [1,2]. The estimate rose to 74% in 2003 based on exon-exon junction microarrays [3] and then all the way to 94% in 2008 by the use of second generation RNA sequencing (RNA-seq) [4]. What was once the exception has now become the norm [5], a fact that may be especially significant given that the human genome contains only a few more genes than *C. elegan*. As highlighted by the ENCODE project [6], RNA splicing is complicated and has called into question the very definition of the gene as a unit of heredity. Since the majority of human genes contain multiple exons and express at least two splice products, gene expression cannot be fully meaningful without considering alternative splicing as well.

It is known that alternatively spliced transcripts of a given gene can code for protein variants with varied biological functions or cellular localizations. Human spliceosome is a complex dance between trans-acting and cis-acting signatures. Regulation, disruption and mutations in any one of these elements has the potential to provide tumor cells with selective advantage during cancer evolution. Numerous gene-by-gene studies have linked splicing to cancer. Genome-wide profiling of splicing in cancer using microarray technologies has revealed that differential splicing may play a key role in cancer progression and metastasis. A comprehensive review of systematic profiling of splicing in various cancer types using genome-wide microarray technologies suggest that differential exon inclusion or skip events may drive cancer and can be used as biomarkers in cancer [7].

The exposure of the entire RNA content within samples by RNA-seq technologies, including novel splice isoforms, has encouraged the development of methods for *de novo* identification of splice events expressed in samples [8-10]. The use of these *de novo* methods has been attractive, because a large number of splice events are believed to be yet unidentified. More recently, these methods are used to discover disease-specific splicing from RNA-seq data [11,12].

Genome-wide profiling of alternative splicing is not new. Before the invent of RNA-seq technologies, genome-wide profiling of RNA splicing in biological samples included exon arrays [13], splice junction arrays [14,15], and genome-wide tiling arrays [16]. Use of these technologies to profile known splicing events in various biological contexts has already revealed the importance of splicing in cancer research. A recent review of genome-wide profiling of splicing in cancer using various microarray platforms suggests that splicing in cancer is prevalent, regulated and that novel therapeutic strategies are emerging [17,18].

The success of microarrays in profiling known splicing in cancer can be extended to identifying tumor specific splicing events in reads from RNA-seq using virtual microarray experiments. In such an experiment, short RNA reads from RNA-seq can be considered virtual equivalent of cellular RNA, *in silico* mapping of reads can be considered virtual equivalent of hybridization and the sequences of exon-exon junction probes equivalent of virtual microarray platform. Hence, a non-redundant reference database of known splice junctions can be used to directly map RNA reads to detect and measure expression levels of known splice events. Although such an approach is limited to detection, by augmenting the database with predicted junctions, one could also infuse discovery into this approach [4].

Here we have profiled less than a million known and predicted splice events to identify tumor-specific splicing in prostate tumor using a RNA-seq dataset of matched tumor-normal from ten individuals downloaded from NCBI public repository.

## Results and discussion

### Validation of SPEventH based prediction

**Figure 1:**
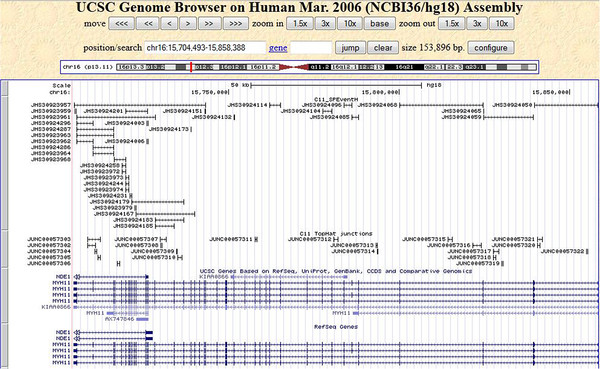
Splice junctions predicted by both SPEventH and Tophat for the gene MYH11 gene in T11 sample.

Since majority of the human genes are multi exon genes, a large number of constitutive splice junctions for highly expressed genes should be expressed irrespective of the splice variants. Methods to predict splice events can be validated by its ability to predict constitutive junctions for highly expressed genes within a sample. To assess the ability of SPEventH to detect constitutive junctions of highly expressed genes, we used ten most highly expressed genes from sample T11 (SRX022067). Table 1 lists the total number of exons in these genes and the number of junctions predicted by both methods SPEventH and Tophat. SPEventH has been able to predict majority of the constitutive junctions. On the other hand, to our surprise, Tophat failed to detect constitutive junctions for any of these genes. Even for MYH11 gene, for which Tophat predicted many junctions, the junctions are found inconsistent with known exon locations for this gene (Figure 1).

**Table 1:** Shows the total number of junctions predicted by SPEventH and Topaht for the top ten most highly expressed genes in T11

**Genes**	**Number of exons**	**SPEventH predicted/represented**	**Tophat**	**Gene expression**
KLK3	5	21/38	0	210099
KLK2	5	23/36	0	135249
MYH11	42	24/115	21	56872
EEF2	15	43/104	0	38561
MSMB	4	21/39	5	25208
LTF	17	15/71	1	36365
FLNA	48	71/151	0	27648
SRRM2	15	34/60	0	23211
ACPP	11	15/34	2	21250
NDRG1	16	43/131	0	20155
MLPH	15	28/72	1	19556
ACTG2	8	17/35	5	18356

SPEventH predictions in column 3 are also augumented with the total number of events represented in the SPEventH database for the respective gene.

**Figure 2:**
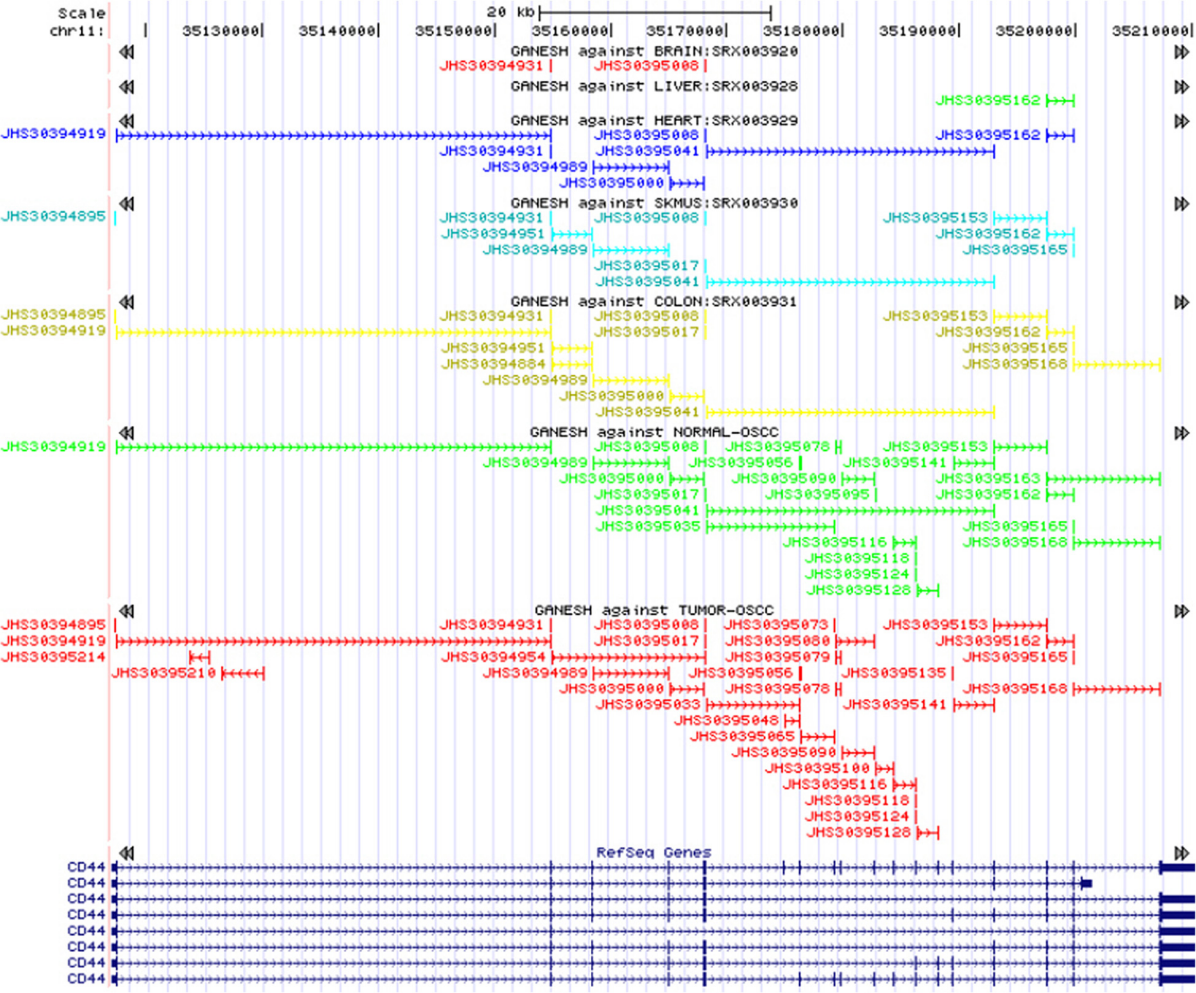
**CD44 splice isoforms, CD44s (JHS30395041) and CD44v1-10 (JHS30395033), in normal and tumor samples respectively as sown on the UCSC genome browser.** Tracks from top are for normal brain, normal liver, normal heart, normal skeletal muscle, normal colon, matched normal from OSCC patient, and matched tumor from OSCC patient.

To test the ability of SPEventH in identifying differential splicing in cancer, we used the splice variants of the gene CD44, which are the most extensively studied for differential splicing in cancer. It is well known that a short variant, CD44s, is ubiquitously expressed in majority of the normal human tissues and a long variant, CD44v1-10, is specific to cancer tissues [19,20]. Short RNA reads from several normal human tissues including brain, liver, heart, skeletal muscle, colon (SRP000302) and matched tumor-normal samples from OSCC (SRP002009) are mapped onto SPEventH using bowtie allowing for two mismatches. At the coverage of sequencing in these datasets, CD44 is not expressed in brain and liver (Tracks A and B of Figure 2). However, in the normal tissues, where the gene is expressed, for example in heart, skeletal muscle, colon and normal OSCC, the wild type variant (CD44s) represented by the probe JHS30395041 is expressed. The Track G of Figure 2 for OSCC tumor sample, the only tumor sample in this Figure, shows expression of the long form CD44v1-10 represented by several probes (JHS30395033, 065, 090, 100, 116, 118, 128, 141). Interestingly, while the variant CD44v1-10 is missing in OSCC normal but expressed in the respective tumor, the CD44s variant is not expressed in OSCC tumor although it is expressed in the respective normal. To our knowledge, this is the first report of tumor-specific differential splicing of CD44 gene in oral squamous cell carcinoma.

### Comparison of SPEventH and Tophat predictions

The numbers of splice events in SPEventH mapped by RNA reads from the twenty samples used in this study, as listed in Table 2, range from 75,000 to 150,000 (Table 3, Column 2). Comparable numbers of splice events are also predicted by Tophat in all twenty samples (Table 3, Column 3). Also, twenty to thirty percent (20-30%) of the splice events in each sample are found to be predicted by both the methods after normalizing for chromosomal coordinates (Table 3, column 4).

**Table 2:** Lists the accession IDs of the 20 samples from 10 individuals used in this study along with read coverage

**Samples**	**Accession ID**	**Depth**
T11/N11	SRX022067/ SRX022085	21993400/29523906
T13/N13	SRX022068/ SRX022086	31061605/29495276
T15/N15	SRX022069/ SRX022087	32535354/28473963
T19/N19	SRX022072/SRX022088	32598639/23807013
T23/N23	SRX022073/ SRX022089	30409693/29323060
T02/N02	SRX022060/SRX022080	10751725/8174610
T03/N03	SRX022061/SRX022081	8100448/8011774
T06/N06	SRX022063/SRX022082	13137600/8002207
T08/N08	SRX022065/SRX022083	8338185/5278786
T09/N09	SRX022066/SRX022084	7551225/5630881

**Table 3:** Shows the number of exon-exon junctions on which reads were uniquely mapped from each sample listed in column 1 by the two methods SPEventH (Column 2) and Tophat (column 3)

**Sample**	**SPEventH**	**Tophat**	**Common**
T02	117076	120864	43891
T03	97898	101032	36109
T06	118972	145448	41827
T08	99557	101261	38229
T09	86033	103793	31510
T11	134996	135378	26818
T13	167250	166213	26758
T15	164348	192518	28383
T19	168447	198934	27790
T23	164983	188546	30043
N02	102874	92880	39396
N03	97750	92234	36411
N06	89427	99376	31770
N08	75131	65474	26844
N09	78205	68111	29759
N11	153994	155169	24175
N13	155390	175640	26132
N15	148237	167555	26825
N19	151464	149138	36617
N23	154390	157457	30576

Last column lists the number of junctions commonly predicted in each sample by both methods.

**Figure 3:**
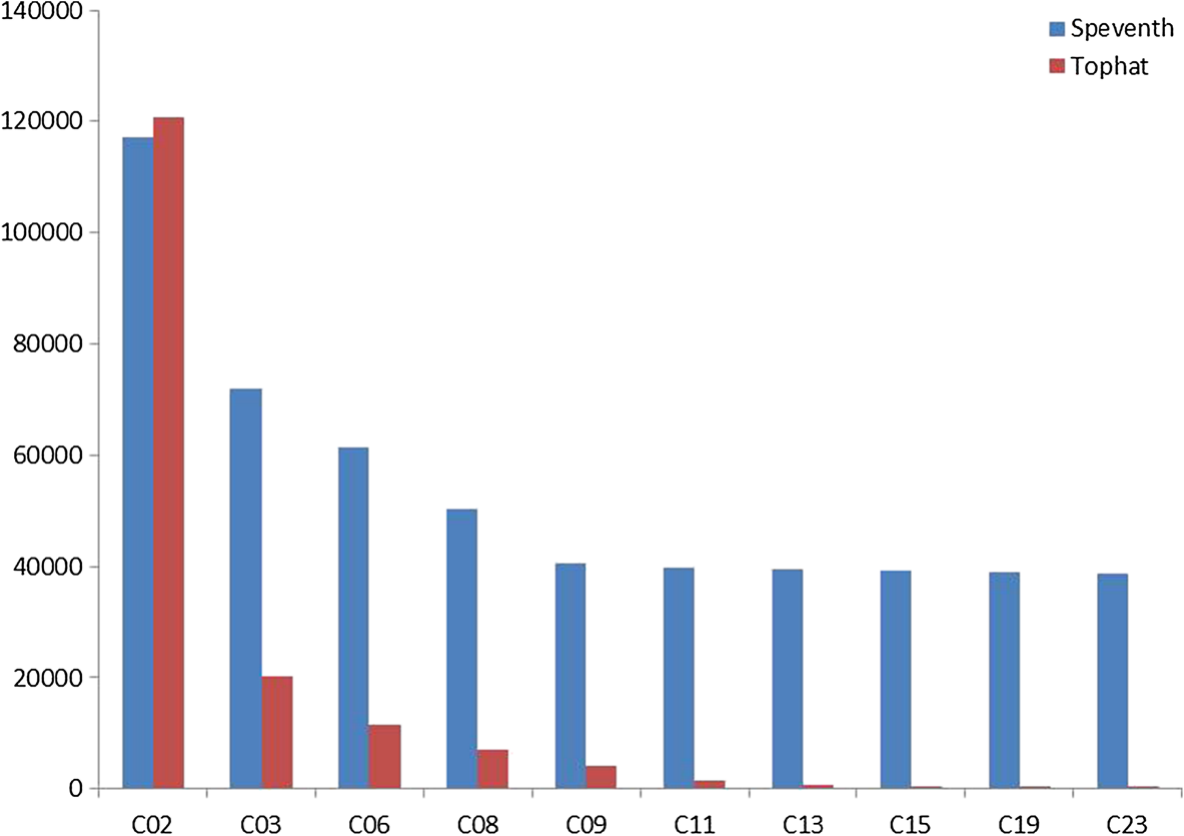
Histogram showing the decline of Tophat and SPEventH hits as we incrementally add tumor samples one by one.

As shown in Figure 3, roughly 40,000 splice events from SPEventH are commonly mapped by all ten tumor samples. Majority of the common events must be constitutive splice junctions of genes expressed in a prostate-specific or tumor-specific manner, which are expected to be expressed irrespective of the splice isoforms. In Figure 3, although the number of common junctions among tumor samples appear to saturate at 40,000 events after addition of 5^th^ or 6^th^ sample, the number of events common to all twenty samples including the ten normal drops to ~20,000 (not shown). This drop is suggestive of the presence of both tumor-specific constitutive and alternative splice events. In comparison, splice events common to all ten tumor samples predicted by Tophat is only 404 (Figure 3), which drops to only 30 events when all twenty samples are compared. This is despite the fact that 25-40% of Tophat events and SPEventH events are found common in individual samples (Table 3, column 4). This could be because Tophat attempts to predict all junctions for each sample *de novo* and many of the constitutive junctions may be predicted in some samples but missed in others. All further analysis is carried out using SPEventH approach.

### Prostate cancer specific splicing from SPEventH

**Figure 4:**
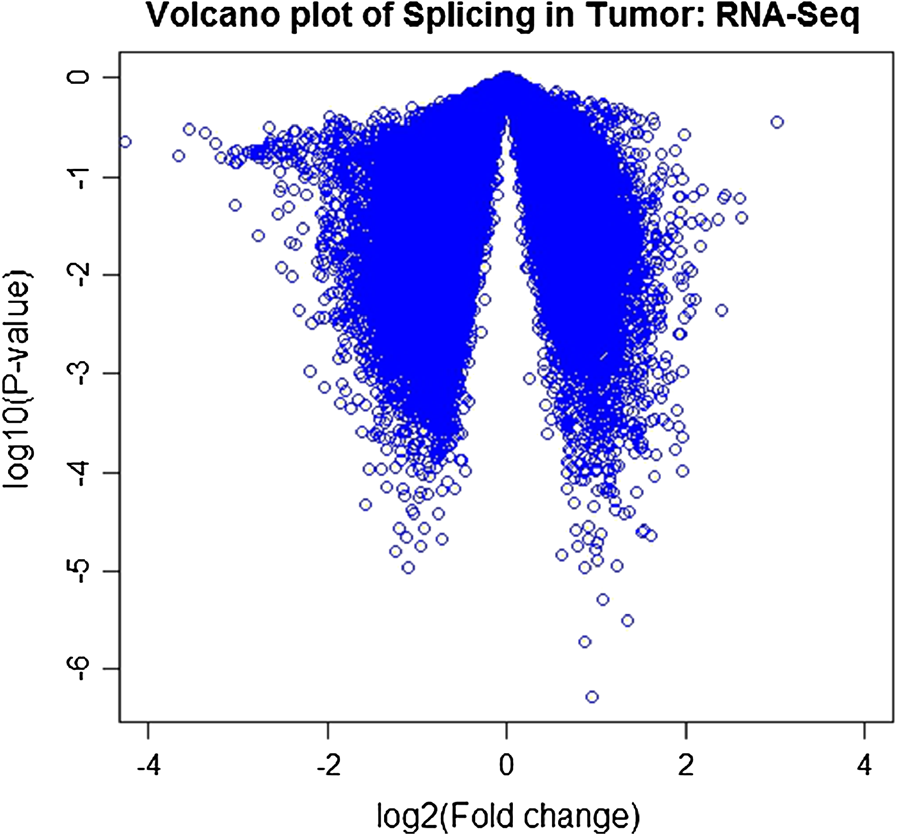
Volcano plot for p-value versus fold change computed using RPKM values for each gene for all 20 samples including 10 matched tumor and normal samples calculated using normal as control.

**Figure 5:**
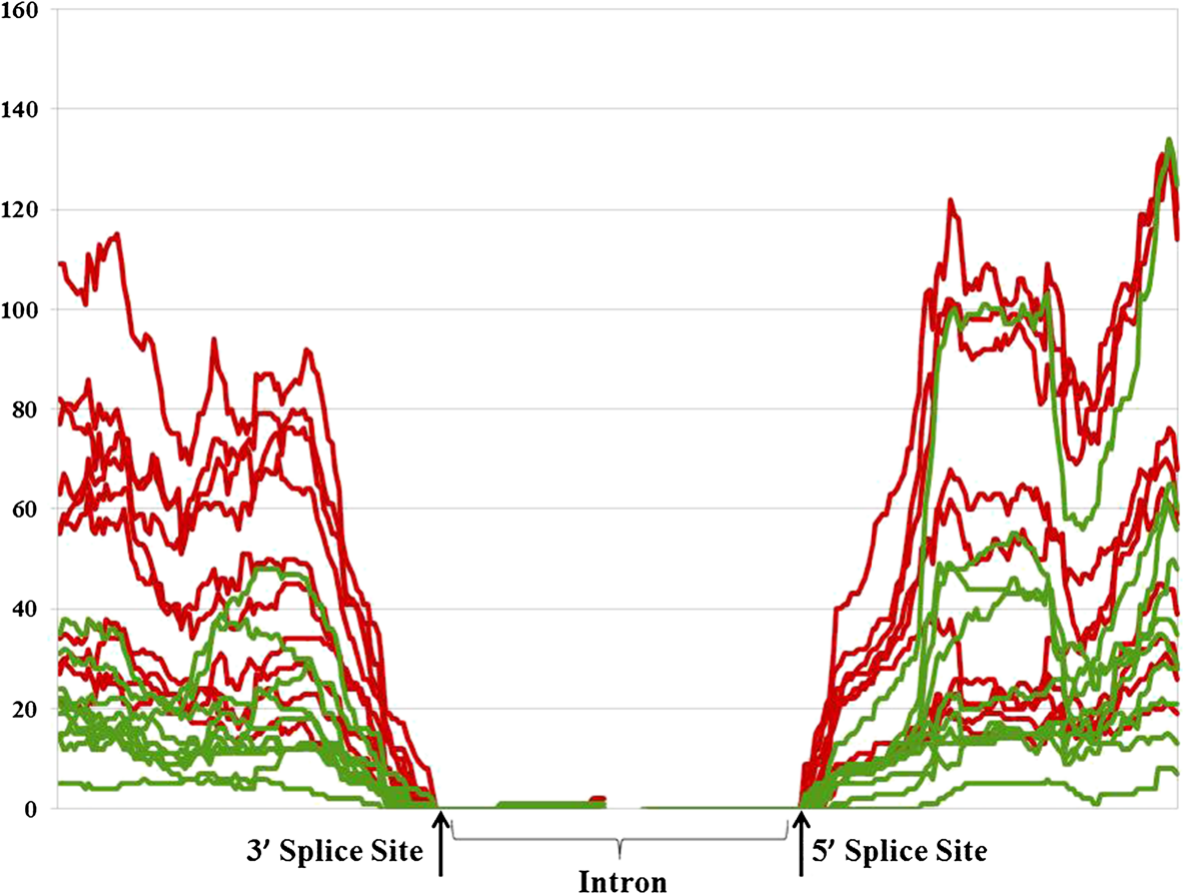
Base level expression around the donor and acceptor sites of a splice junction from gene PPP3CA gene in all 20 samples.

Figure 4 shows the volcano plot of SPEventH-based analysis using R-package for the 20 matched tumor-normal samples from the ten individuals. Table 4 lists the 61 splice events that are up- and down regulated in a tumor-specific fashion with a p-value of <0.0001 and a fold change of > 1.5. Forty-one of these 61 events are validated by measuring base level expression on either side of the junctions as shown in Table 4, Column 7. Figure 5 shows base level expression of one of the junctions from the gene PPP3CA across all 20 samples. Table 5 lists PCR primers including amplicon size for all events listed in Table 4 except for those that had no evidence in public transcriptome databases.

**Table 4:** Splice events up-and down-regulated in a tumor specific fashion with a p-value of < 0.0001

**Sl.No**	**Gene name**	**Source of evidence**	**Are they from alt. splicing region?**	**P-value**	**Fold-change (log2)**	**Base level expression around splice sites**
1	TARP	BC105589.J1-2	TRUE	9.08E-05	1.65501	✓
2	SH3RF1	NM_020870.J11-12.	FALSE	2.32E-05	1.602221	-
3	PPP3CA	NM_000944.J5-6	TRUE	2.60E-05	1.52093	✓
4	MARCH6	NM_005885.J9-10	TRUE	2.48E-05	1.501025	-
5	ARFGAP3	NM_014570.J2-3	TRUE	6.28E-05	1.441878	✓
6	TAOK3	NM_016281.J18-19	FALSE	4.00E-05	1.379249	✓
7	EIF2AK4	NM_001013703.J37-38	TRUE	3.15E-06	1.346129	-
8	TMEM87A	NM_015497.J7-8	TRUE	3.81E-05	1.318233	✓
9	TOM1L1	NM_005486.J5-6	TRUE	1.14E-05	1.222369	✓
10	FERMT3	NT_033903.311.J10-11	FALSE	5.09E-05	1.211549	-
11	RPL7L1	NM_198486.J2-3	TRUE	4.14E-05	1.200866	-
12	TTC3	NM_003316.J18-19	TRUE	6.18E-05	1.183933	-
13	ACSL3	NM_004457.J12-13	TRUE	6.56E-05	1.158874	✓
14	SF3B1	NM_012433.J4-5	TRUE	8.85E-05	1.147913	✓
**15**	**KLK3**	**BF372485.J1-2.**	**TRUE**	**8.13E-05**	**1.14738**	**-**
16	CTBP2	NM_001083914.J2-3	TRUE	6.29E-05	1.114432	✓
17	SR140	NM_001080415.J25-26	TRUE	6.82E-05	1.109745	-
18	GTF3C1	NM_001520.J29-30	FALSE	5.11E-06	1.080653	-
19	CHEK1	NT_033899.609.J6-7	TRUE	6.83E-05	1.071581	✓
20	C15orf44	NM_030800.J11-12.	TRUE	2.45E-05	1.058924	-
21	CCDC14	NM_022757.J6-7	TRUE	1.26E-05	1.018248	-
22	KIAA0368	NM_001080398.J30-31	TRUE	6.62E-05	1.009773	✓
23	COPZ1,MIR148B	NM_016057.J3-4	TRUE	1.92E-05	1.001943	✓
24	ECH1,HNRNPL	NM_001533.J4-5	TRUE	1.64E-05	0.991637	✓
25	CNDP1	chr18.71.004.a.J2-3	TRUE	4.40E-05	0.97475	-
26	NULL	CN480760.J3-4	FALSE	5.32E-07	0.954029	✓
27	BCLAF1	NM_014739.J8-9	TRUE	2.87E-05	0.905132	✓
28	SON	NM_138927.J10-11	TRUE	2.17E-05	0.902497	✓
29	HSP90B1	NM_003299.J11-12	TRUE	1.11E-05	0.881922	✓
30	TTC19	NM_017775.J3-4	TRUE	8.24E-05	0.87852	-
31	TSPAN1	NM_005727.J6-7	TRUE	9.96E-05	0.873753	✓
32	NET1	NM_001047160.J6-7	TRUE	1.90E-06	0.869833	✓
33	ATXN10	NM_013236.J4-5	TRUE	8.27E-05	0.826939	✓
34	CANT1	NM_001159772.J3-4	TRUE	1.80E-05	0.793932	-
35	CCDC53	NM_016053.J3-4	TRUE	2.60E-05	0.780735	-
36	HADHA	NM_000182.J7-8	TRUE	8.58E-05	0.758566	-
37	CBS	NT_030188.38.J3-4	FALSE	4.96E-05	0.745529	-
38	SLTM	NM_024755.J4-5	TRUE	6.69E-05	0.675305	✓
39	DDX5	NM_004396.J3-4	TRUE	9.69E-05	0.671894	✓
40	HSPA8	BG699643.J2-3	TRUE	1.46E-05	0.609402	✓
41	LYNX1	NM_023946.J2-3	TRUE	4.67E-05	−1.56988	✓
42	KLHL29	NM_052920.J10-11	FALSE	7.16E-05	−1.33184	✓
43	ITGA3	NM_002204.J17-18	TRUE	1.59E-05	−1.23949	✓
44	CNN2	NM_004368.J6-7.	TRUE	2.77E-05	−1.19031	✓
45	SIN3A	NT_010194.1124.J12-13	FALSE	6.71E-05	−1.15772	✓
46	LAMA5	NM_005560.J78-79	FALSE	5.75E-05	−1.13839	✓
47	DPM2	NM_003863.J2-3	TRUE	2.18E-05	−1.11538	✓
48	PINK1	NM_032409.J6-7	TRUE	1.10E-05	−1.09024	✓
49	NCAPD2,SCARNA10	NT_009759.130.J28-29	FALSE	4.16E-05	−1.06536	-
50	EML2,MIR330	NM_012155.J17-18	FALSE	3.73E-05	−1.04227	✓
51	HEATR7A	NM_032450.J42-43	TRUE	5.59E-05	−0.97289	-
52	HEATR7A	NM_032450.J18-19	TRUE	1.80E-05	−0.96627	✓
53	ARAP1,STARD10	NM_001040118.J12-13	FALSE	6.18E-05	−0.95743	✓
54	ALS2CL	NM_147129.J15-16	FALSE	9.60E-05	−0.94678	✓
55	RAB25	chr1.155.009.a.J2-3	FALSE	2.75E-05	−0.91566	✓
56	NULL	NT_030188.20.J5-6	FALSE	5.90E-05	−0.88101	-
57	ATF6B,TNXB	NM_019105.J22-23	FALSE	9.19E-05	−0.85967	✓
58	RUVBL2	NM_006666.J11-12	FALSE	3.78E-05	−0.75383	✓
59	MAP3K12	NM_006301.J4-5	TRUE	6.44E-05	−0.72161	✓
60	AIFM2	NM_032797.J7-8	FALSE	2.12E-05	−0.72049	✓
61	ATP2A2	NM_001681.J19-20	FALSE	7.93E-05	−0.66293	✓

**Table 5:** PCR primers for events for which a transcript sequence was available in the public databases

**Sl. no**	**SpliceJunctionID_GeneName**	**Amplicon**	**Forward primer**	**Reverse primer**
1	JHS32215053_TARP	119	TCCCGGAACAAAGCTTATCA	AAGACCAGGGTTCCAGTCCT
2	JHS31939933_SH3RF1	113	AGTTCCCATCGCTCCACCT	GAGGAGGATAGGAAACCACCA
3	JHS31912679_PPP3CA	106	GTGTGTGCATGGTGGTTTGT	CAGGATATCACACATAGGTCCA
4	JHS31958799_march6	108	GGGAAAGAATGCTAGGACTTGA	GGTAAGGGCAAAATGCAAAA
5	JHS31704238_ARFGAP3	121	GGGCAAGCATAACCTATGGA	TGAAACCATGACCAGTTGGA
6	JHS30608278_TAOK3	132	AAAGCATGTCATGGAACTTCG	TCCAACTGGTGATTCTTGAGTG
7	JHS30807650_EIF2AK4	103	TGAAATTCTGGCTGTGGATCT	CAGCTGCTTCACAGTTGTGTT
8	JHS30813189_TMEM87A	114	TGGCATTTCATCCTCAAAGG	AAGGGATAGTCTTCAAGTGTGAGG
9	JHS31115850_TOM1L1	117	CAGAAGCAGAGGCTGAAACA	CAGTCGAGTTCTTTGGAGCA
10	JHS32114870_RPL7L1	148	CGGAGCAAGAGCAAAGAAAA	CGCTTAAACCTGAGCCCTTT
11	JHS31615632_TTC3	110	GCTGAACGGTTTAGATCCTCA	CAGGCTGTCCTATTCCAAGG
12	JHS31498135_SF3B1	116	AGCATAGGCGGACCATGATA	TTGTTCTCGCATTACATCCA
13	JHS31332823_KLK3	146	ACAGCTGAGCCACTCTGAGG	CAGGGGTCGGGGAGATATAG
14	JHS30347840_CTBP2	106	TCCCTTAAAAAGACGGACAGC	AGGGGAACTTGCAGGAGTCT
15	JHS31820386_SR140	100	GCCAGAGTTTTCAGGAGCAA	TCTTTCTTGTCTCTTTCTCGTTCTC
16	JHS30945721_GTF3C1	123	GGTGCTTCAGAACCTGATCC	AAGGCCTTCACAAGAACGTG
17	JHS30838314_C15orf44	131	GCCTGCAGACAGATGTACAGA	TTTCAGCAGGTCCAGGAAAC
18	JHS31803136_CCDC14	125	TCTCAGGCAACTCCTCATTCT	GCAAACTGACTGTTGCCATC
19	JHS32473823_KIAA0368	107	GGAGAGACAGTGGTATTTCAAGG	GGCTGGCTAAGATCACTTGC
20	JHS30556221_"COPZ1,MIR148B"	107	GACGACACCTACCCCAGTGT	ACTGTCAGGCCTTCCAAGAG
21	JHS31292790_"ECH1,HNRNPL"	104	AGAATGGAGTTCAGGCGATG	CAGAGTGCAACAGCCAGAATA
22	JHS32163374_BCLAF1	107	CCAAGTACCCTGAGGAAGCA	CGAAGGTCAGCAGAGTCACA
23	JHS31612151_SON	126	GGGAACTTCTCTGCTGCAAT	ATCAGGGCCACTATCATGGA
24	JHS30594323_HSP90B1	118	GTCAAGGGTGTGGTGGACTC	TCATGTCCAGCGTTTTACGA
25	JHS31048200_TTC19	103	ATTTTGCATGACGCTCTTCG	GCTGACCCCGTATAAATGCT
26	JHS30077792_TSPAN1	103	CAAGTGTGGAACACCACCAT	GGGGAAAGGCACTGTTCTCT
27	JHS30255522_NET1	101	CCCGAGGTGAACAGGATTTA	TGTGAGTTCCTCTTCTGACATGA
28	JHS31707588_ATXN10	130	ATTGTTTGGGTGCATGCTTT	CCTCCAGTTCTTTCATTCTTTCA
29	JHS31151584_CANT1	116	TGCTGTGGAAAGACTTCACG	ATCCGGAGGGAGTGCATAG
30	JHS30592849_CCDC53	112	TGAGGAGAAACTGGCAGACC	TTCAACTGTGACATCATCTAGGC
31	JHS31375495_HADHA	115	TGGACATGATGCTGACTGGT	GTCCGTTCCTCTGGAGGTTT
32	JHS30830235_SLTM	113	TCTGCAGAAGAAAACAAGAGAGC	TCATCTTCACCTTCTTGAGCTTC
33	JHS31129340_DDX5	124	GCCCGAAGCCAGTTCTAAAT	CTTAGAGCAACTGGCCATCC
34	JHS30485155_HSPA8	109	AGTAGCAATGAACCCCACCA	CACCATAAAGGGCCAATGTT
35	JHS30035592_PINK1	123	CGGAAACGGCTGTCTGAT	AGCCCGAAGATTTCATAGGC
36	JHS31111518_ITGA3	117	CTGCACACAAGGGACCTTCA	GCTAAGCGAGGTCTGGAGTG
37	JHS32407856_HEATR7A	112	GTCAGCATTGGGAAACGACT	TCTCGCAGGAACATCAACAG
38	JHS30295263_AIFM2	123	AAGATCAACAGCTCCGCCTA	ACAGTCACCAATGGCGTAGA
39	JHS32488635_DPM2	134	TACTACACCGCCTGGGTGAT	AGTCCCACAAACAGGAGCAG
40	JHS31214980_CNN2	116	AGATGGGCACGAACAAGTG	CAGGGACATGGAGGAGTTGT
41	JHS31314031_"EML2,MIR330"	118	GAATGGGCCACAGCTACTTG	AAGCCAGCAACTTCCCATC
42	JHS31324130_RUVBL2	119	CCTGATCATGGCCACCAA	TGTAGGGGGTGGTGGAGAC
43	JHS32401588_LYNX1	128	CTGATCCTGGTGGTCCTCAT	GTGGTCATGCAGTAGGCAAC
44	JHS32407984_HEATR7A	136	TCAAGAGCAGCTGGGAGAAC	TCCTTCAGCAGGATCTGGAG
45	JHS31597126_LAMA5	125	GGGAGTTCTCCACGTCAGTG	CACGGTGTGGTTGCTCTG
46	JHS30447530_"ARAP1,STARD10"	104	TCAACCTCTGTGTTGTTATCTGC	CCACACCTTCCTGTCCATCT
47	JHS30554360_MAP3K12	107	AGCACCCCAACATCATCACT	AGCCCGCAGTACCTCATACA
48	JHS31372783_KLHL29	100	GTAGTGAGTGCAGGGGACAAC	TCCAGTTATCAAGCAGGGACA
49	JHS30600491_ATP2A2	106	TGTCACTCCACTTCCTGATCC	GGCAAGGAGATTTTCAGCAC
50	JHS32096926_"ATF6B,TNXB"	121	CTGGGCGCAAGTACAAGATG	GTCATGGTAGGCACTGCTTG
51	JHS31749149_ALS2CL	111	ACCTTCACCAGGGACCTGAC	CTGTGTGTGCAGTCCTCTCC

**Figure 6:**
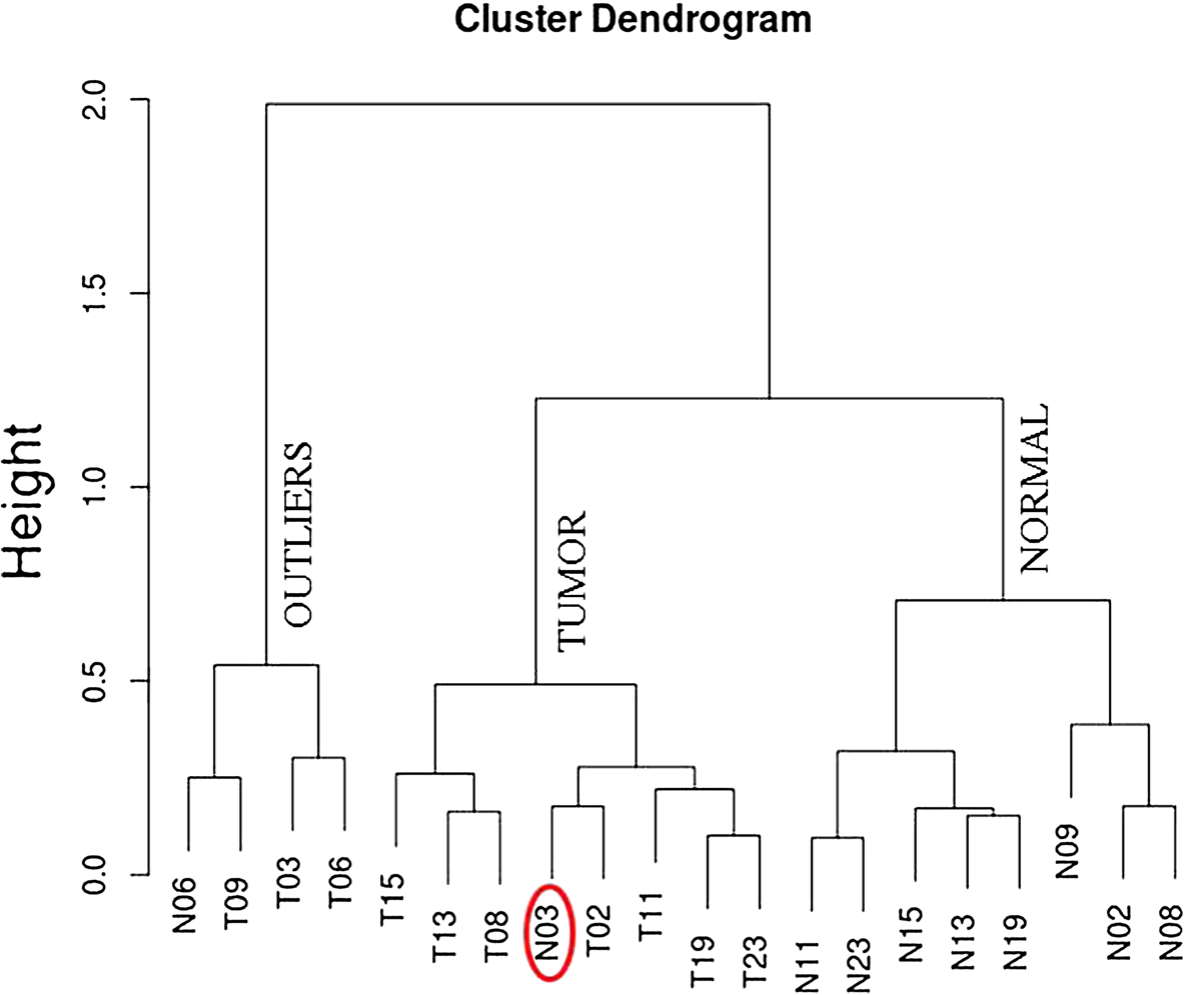
Clustering of all 20 samples using all the 765 splice events with p-value <0.001 and a fold change of >1.5.

The p-value cutoff used to generate Table 4 is rather very stringent. In order to cross check the significance of the 765 events with p-values of less than 0.001 and a fold-change of >1.5, the twenty samples are clustered using RPKM values for these events in all 20 samples. As shown in Figure 6, there are three major clusters including two clusters representing tumor and normal. The normal cluster includes eight (8) normal samples. The tumor cluster includes seven (7) tumor samples and one normal sample of low sequence coverage. All the samples in the outlier cluster are low sequence coverage.

**Figure 7:**
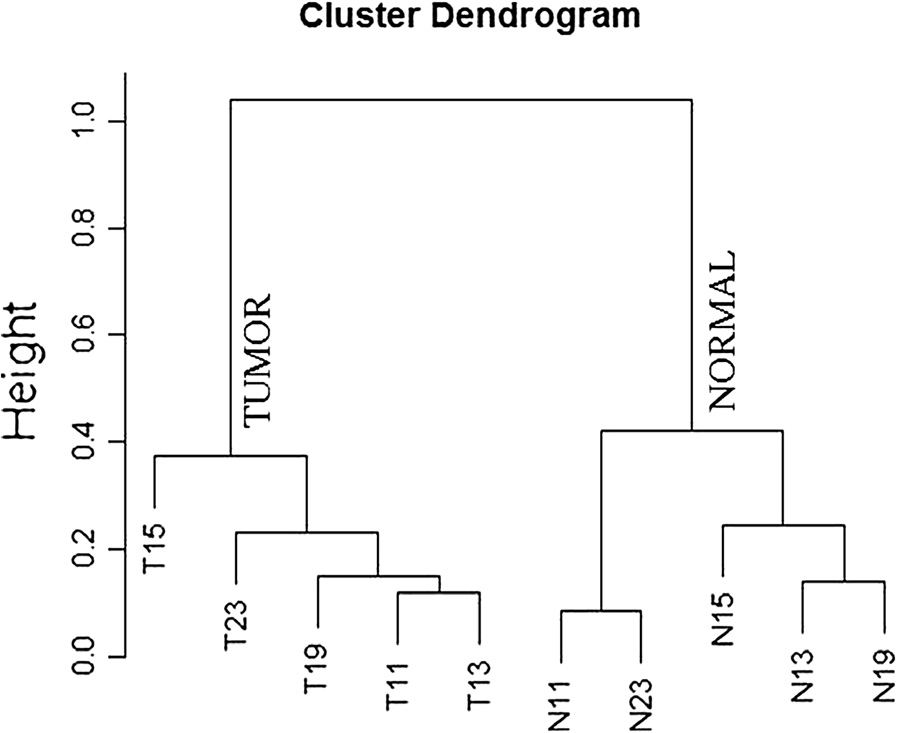
Clustering of ten samples from the patients with high sequence coverage using the 672 events with p-values of <0.001 and a fold change of >1.5 left after removing 93 events that may have been from differential gene expression.

One of the challenges in the identification of tumor-specific splice variant is to filter out splice events from differentially expressed genes in tumor. To identify such splice events among the 765 events, tumor-specific gene expression for the corresponding genes across the twenty samples are computed. Splice events from genes displaying tumor-specific expression levels with p-values <0.01 are considered as resulting from overall gene expression changes. Out of the 765 events a total of ninety-three (93) events are found to be belonging to genes that are considered differentially expressed. Figure 7 shows results from clustering the ten samples with high sequence coverage using the RPKM of the 672 remaining splice events. Figure 7 shows two tight clusters of normal and tumor samples. Note that since all the samples in the outlier cluster in Figure 6 are samples with low coverage, only the ten samples from the five individuals with high sequence coverage are used.

In order to computationally validate the findings with samples from other individuals, not included during the discovery process, we profiled splicing in another RNA-seq dataset independently generated by another group (SRP003611) [21]. Two splice events in genes PPP3CA and SLC20A2 are found to be significantly up- and down-regulated in both datasets with a p-value of less than 0.001 in both sets (shown bold in Table 4). This is despite the fact that the sample preparation protocols for both datasets are different. Also, the only evidence in the public EST transcript database, (BF372485), for a tumor-specific splice event connecting an intron from KLK3 gene to that of KLK2 gene is derived from prostate tumor.

## Conclusions

Deep sequencing of RNA provides a promising means of understanding the role of alternative splicing in cancer. A reference database of splice events, such as SPEventH, provides a useful tool to expedite the analysis of RNA-seq data, while also providing a ready link between raw data and existing body of knowledge necessary for biological interpretation and downstream validation. To our knowledge SPEventH database is the only splice event database that includes splice junctions resulting from alternative 5’ and 3’ events, a class of splicing that is also prevalent, and understudied, in human. The key attributes of this database are high-confidence, non-redundancy, detailed annotations, and simple format for ease of use.

We have demonstrated the value of SPEventH in the identification of prostate tumor specific splicing from RNA-seq datasets. We have identified a large number of tumor-specific splice events in prostate cancer and have authenticated the findings by computing base-level expression immediately around the donor and acceptor sites for each differentially expressed splice event. Also, the significance of the hundreds of splice events with p-values less than 0.001 was addressed by clustering the 20 samples using the RPKM values for these events. Our observation is that despite normalizing for sequence coverage using RPKM, samples with low coverage could not be clustered using the signature events. This suggests that for profiling splicing at least 30–40 million reads may be necessary.

Here, RNA-seq datasets generated by two independent groups have been compared to validate the findings. We find that the most significant events from the two datasets are quite divergent, suggesting either heterogeneity in the cancer types or differences in sample preparation protocols by the two groups. Since many of the most up-regulated genes from the validation dataset (SRP003611) had many snoRNAs ( [21]) than from the discovery dataset (SRP002628), we believe that the validation set may not have been selected for protein coding RNAs. Despite such discrepancies, we found two splice events from genes PPP3CA and SLC20A2 that are significantly up- and down-regulated in a tumor–specific fashion.

Identification of tumor-specific splice events is complicated by the expression of a large number of constitutive junctions from differentially expressed genes. In order to separate those events that are purely from differential splicing, all events from differentially expressed genes were removed from the final signature. It is likely that many differential cancer driving splice events from differentially expressed genes may have been removed by this crude approach. Better methods are needed to address this issue.

This is perhaps one of the first efforts to compare the performance of a de novo splice prediction method such as Tophat to a splice detection method such as SPEventH in the identification of tumor-specific splice signatures. The *de novo* method like Tophat is considered attractive for their capacity to discover novel events. However, we see that Tophat performs poorly in predicting known splice events including constitutive junctions of highly expressed genes consistently across large number of samples. On the contrary, detection methods like SPEventH are not only sensitive for known events but are amenable for comparison across large number of samples. Also, by augmenting predicted splice events from gene prediction algorithm, discovery is also built into SPEventH-based profiling of splicing across large number of samples. With advancing sequencing technologies, improving bioinformatics tools, and proliferating public data sets a reference database of annotated splice events in human will mature and will become critical for profiling alternative splicing in biological samples.

## Materials and methods

Results reported in this manuscript did not involve any experimental work on human or animal samples. All reported findings are obtained by bioinformatics analysis on the data downloaded from NCBI repository of Short Read Archive as listed in Table 2.

### Construction of SPEventH database

A reference splice event database, SPEventH, containing probes of optimal length representing exon-exon and exon-intron junction sequences for 731,954 splice events is derived from a database of non-redundant splice junctions in human, SEHS1.0 [22], which is validated for genome-wide profiling of alternative splicing using microarray technology [23]. The SEHS1.0 was created using the alignment of 8.5 million transcripts including 8,089,335 ESTs, 287,440 mRNAs, 34,389 Refseq, 66,803 known genes and 99,128 predicted genes onto the hg18 assembly of the human genome [24]. Construction of SEHS1.0 involved parsing and processing of 8.5 million aligned full-length and partial transcript sequences, identifying the splice sites, applying an alignment quality filter, making the set non redundant, selecting sequences spanning the splice sites, and attaching detailed annotations to the results. The quality filter has removed misalignments arising from low sequence quality. Key parameters enforced that each splice site span canonical donor and acceptor motifs; however, splice events with multiple sequences as evidence were included regardless of the intron motifs. Uniqueness of a splice site was defined by the intron start and end coordinates. Annotations include the data source (such as RefSeq or GENSCAN), list of accessions of sequences that provide evidence for each splice event, intron donor and acceptor motifs, as well chromosomal coordinates and gene symbols.

The probes in the SPEventH database are of length 56 bases including 28 bases from either exons participating in the junctions. This length is optimized to reliably exclude mapping of RNA reads of length 36 base pair, which do not span a junction. Meaning, when 36mer reads of length 36 bases are mapped to probes containing 28 bases on either side of the junction, minimum mapping of 8 bases into the adjoining exon is ensured even for reads mapped staring at position one of the probe or ending at position 56 of the probes.

**Figure 8:**
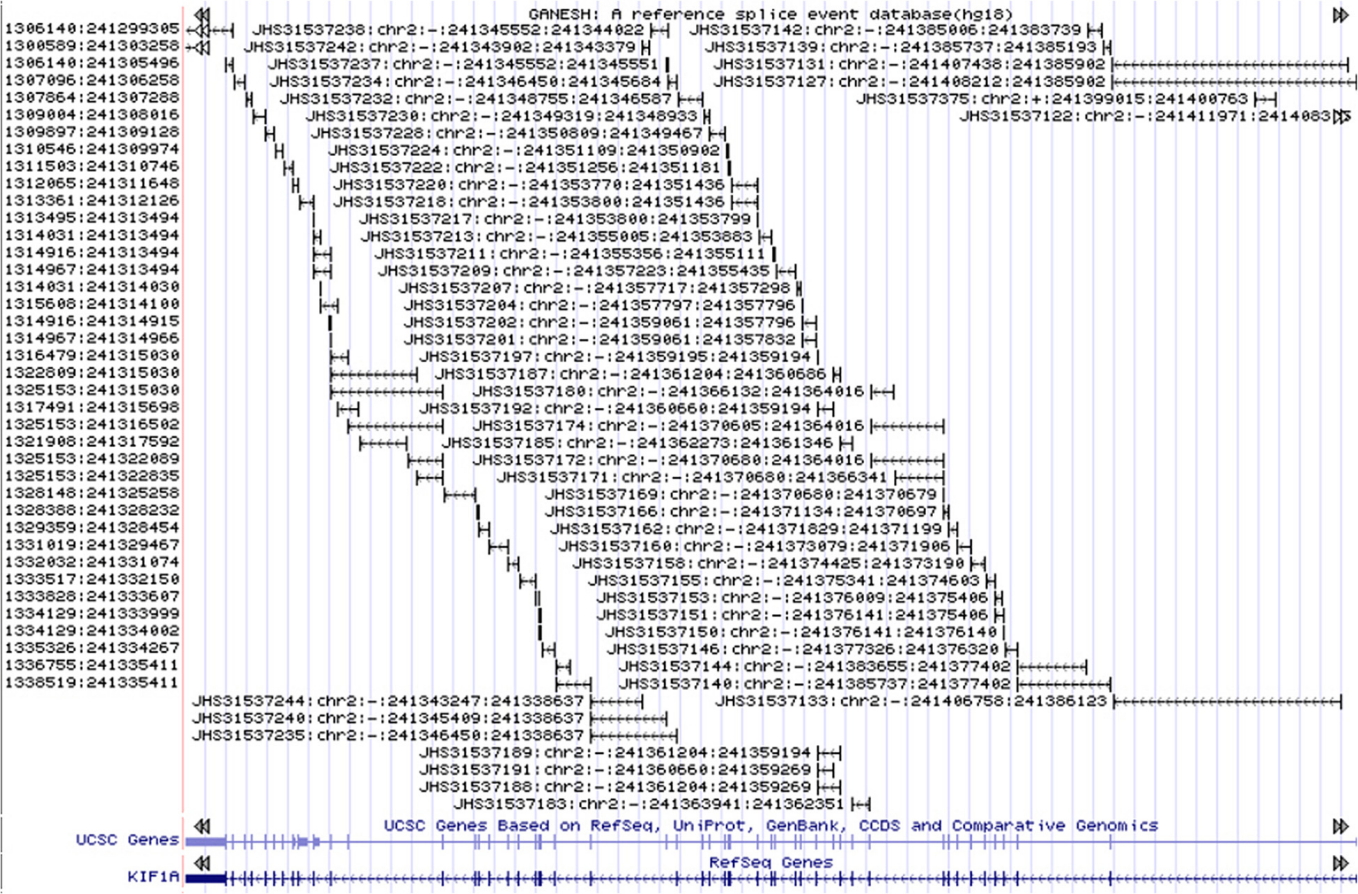
**An anecdotal example of all splice junctions represented in SPEventH database for the gene KIH1A shown on UCSC browser.** The two vertical bars in each fat ‘H’ are exons connected by introns.

Owing to the varying sequence quality of the underlying data sources, splice events in SPEventh differ in evidentiary support. Approximately 58.2% of splice events in SPEventH have experimental evidence in the public sequence repositories. Spot checks of individual genes such as kinesin 1A against the UCSC genome browser provides quality assurance for the completeness and accuracy of the data in SPEventH (Figure 8). Although kinesin 1A contains 46 exons, according to its RefSeq transcript, SPEventH reveals at least 50 exons, including ESTs and predictions, and 91 distinct splice events. SPEventH is available for download as a FASTA file with annotations stored as key/value pairs in the header line (http://resource.ibab.ac.in/SPEventH/). A BED file from SPEventH can also be downloaded from the same site. The count of each splice event in the BED format corresponds to the number of public sequences providing evidence for the respective splice event.

### Mapping short RNA reads to SPEventH

The dataset of short RNA reads derived from 20 samples including tumor and matched normal prostate tissues from 10 individuals is downloaded from NCBI repository with accession IDs listed in Table 2. Tens of millions of short reads from each sample are mapped to junction spanning probes in SPEventH using bowtie. Two mismatches are allowed while mapping and only uniquely mapped reads are considered. Parsing the bowtie output was done by uploading the bowtie fields on to MySQL database, creating one table per sample. Using a SQL query, number of reads mapped to each SPEventH junctions was computed and stored in another table. A mega table is then generated from the twenty individual tables that includes read counts of all the splice events in the database of all samples used in the study (SRP002628). The read counts are normalized across samples by computing RPKM values. The p-value and fold change (log_2_) are computed by comparing RPKMs for normal and tumor for all events in SPEventH using R statistical package. A reduced table of events with p-value <0.001 for all normal and tumor samples is created. A hierarchical clustering of all 20 samples based on the RPKM values for selected events in each sample is performed.

### Mapping short RNA reads to hg18 assembly

Tophat program, version 1.1.2, with default parameters is used to map reads from all 20 samples onto hg18 genome assembly. The default parameters of Tophat and the genome assembly versions are consistent with mapping of reads to SPEventH. The junction coordinates are normalized to those of SPEventH for comparison by using a perl script and MySQL database.

## Abbreviations

SPEventH: SPlice Events in Human; BED: Browser Extensible Data format; RPKM: Reads Per Kilobase of exon model per Million mapped reads.

## Competing interests

Authors have no competing interest.

## Authors’ contributions

AP discovered and validated prostate specific splice events from RNA-seq data. MV has computed exon, gene and base level expression in samples. JB created the code that enabled the creation of SPEventH. RS performed statistical calculations using R package. SS is the Principal Investigator of this project who conceived of the SPEventH database and its use with RNA-seq dataset. She designed the experiment and wrote the first draft of the manuscript. All authors read and approved the final manuscript.
